# Human respiratory syncytial virus genetic diversity and lineage replacement in Ireland pre- and post-COVID-19 pandemic

**DOI:** 10.1099/mgen.0.001379

**Published:** 2025-03-17

**Authors:** Alan Rice, Gabriel Gonzalez, Michael Carr, Jonathan Dean, Emer O’Byrne, Lynn Aarts, Harry Vennema, Weronika Banka, Charlene Bennett, Siobhán Cleary, Lisa Domegan, Joan O’Donnell, Maureen O'Leary, Stephanie Goya, Lance Presser, Adam Meijer, Greg Martin, Hirofumi Sawa, Allison Waters, Cillian De Gascun, Daniel Hare

**Affiliations:** 1 UCD National Virus Reference Laboratory, University College Dublin, Dublin, Belfield, D04 E1W1, Ireland; 2 Institute for Vaccine Research and Development, Hokkaido University, Sapporo, Hokkaido 001-0021, Japan; 3 International Collaboration Unit, International Institute for Zoonosis Control, Hokkaido University, Sapporo, Hokkaido 001-0020, Japan; 4 Centre for Infectious Disease Control, National Institute for Public Health and Environment, Bilthoven, Netherlands; 5 Health Protection Surveillance Centre, Dublin, Ireland; 6 Department of Laboratory Medicine and Pathology, University of Washington, Seattle, USA; 7 Health Improvement, Health Service Executive, Dublin, Ireland; 8 Irish Blood Transfusion Service, National Blood Centre, Dublin, Ireland; 9 UCD School of Public Health, Physiotherapy and Social Science, University College Dublin, Dublin, Ireland

**Keywords:** genetic diversity, human respiratory syncytial virus, lineages, orthopneumovirus, whole-genome sequencing

## Abstract

Human respiratory syncytial virus (HRSV) is a common cause of lower respiratory tract infections globally, and changes in viral epidemiology have been observed in many jurisdictions following the coronavirus disease 2019 (COVID-19) pandemic. Newly licensed vaccines and monoclonal antibodies are anticipated to alleviate the burden on healthcare systems, though such interventions may exert selective pressures on viral evolution. To evaluate the diversity of HRSV in Ireland pre- and post-COVID-19 pandemic, whole-genome sequencing was performed on HRSV-A (*n*=123) and -B (*n*=110) samples collected from community and hospitalized cases, during three HRSV seasons between 2021 and 2024. Additionally, G gene sequences, from HRSV-A (*n*=141) and -B (*n*=141), collected in the 2015–2019 period were examined. Lineages were assigned by phylogenetic analyses including reference lineages. Phylogenetic trees inferred with the G gene and whole genomes were consistent. Changes in the prevalence of certain lineages post-COVID-19 reflected the impact of non-pharmaceutical interventions (NPIs) introduced to reduce severe acute respiratory syndrome coronavirus 2 transmission, with A.D.1 and A.D.5 the dominant HRSV-A lineages and B.D.E.1 the most prevalent HRSV-B lineage. Similar trends were observed in HRSV lineages circulating across Europe during this time. The emergence of a new lineage was identified as a descendant from A.D.1, with eight distinctive substitutions in proteins G, F and L. Other circulating lineages with aa substitutions were observed in the F glycoprotein, which could impact nirsevimab binding. We provide the first comprehensive analysis of HRSV genomic diversity and evolution in Ireland over the last decade and the impact of the NPIs introduced during the COVID-19 pandemic. This study provides a foundation for future public health surveillance employing pathogen genomics to enable an evidence-based assessment of the impact of pharmaceutical interventions on HRSV evolution and disease severity.

­

**Impact Statement**

We provide the first comprehensive evaluation of human respiratory syncytial virus (HRSV) genomic diversity in Ireland, including a detailed analysis of circulating lineages prior to, and following, the coronavirus disease 2019 (COVID-19) pandemic – correlating viral evolution with altered epidemiological patterns after the relaxation of population-scale non-pharmaceutical interventions. In demonstrating significant changes in both the diversity and abundance of viral lineages, coupled with the identification of a novel HRSV-A lineage, we underline the importance of robust whole-genome sequencing (WGS)-based surveillance in the context of planned novel HRSV immunization strategies, targeting the pre-fusion glycoprotein. Conversely, the consistency in phylogenetic topology inferred from G genes and whole genomes facilitated meaningful comparison with pre-COVID-19 HRSV datasets, highlighting the valuable role that partial-genome sequencing can still play for lineage classification in settings with limited access to WGS. Finally, through our description of matching viral evolutionary trends across the European region, we place Irish data in an international context and provide an important baseline for ongoing genomic surveillance of HRSV and prospective evaluation of protective countermeasures.

## Data Summary

Details on supplementary materials are available online at https://doi.org/10.5281/zenodo.12797565.

## Introduction

Human respiratory syncytial virus (HRSV) is one of the most common causes of acute lower respiratory tract infection in young children, with a reported incidence of >33 million cases globally per annum [1]. Preterm neonates, infants less than 1 year of age, the elderly and the immunocompromised are recognized as the cohorts most susceptible to severe HRSV disease, although reinfections in older children and adults with milder presentations also place a significant burden on economic productivity and healthcare systems worldwide [2,3]. Vaccination has been made available and recommended for adults aged ≥60 years [4] and for pregnant women during weeks 32–36 gestation to afford protection to infants during the first 6 months of life [5] in some European countries and the USA, whilst passive immunization with monoclonal antibodies, designed to prevent the development of severe HRSV disease, is available for prophylactic administration in vulnerable paediatric cohorts. This includes palivizumab (Synagis) and, more recently, the long-acting intramuscular preparation, nirsevimab (Beyfortus) [6,7], which was offered in Ireland to all infants born between 1 September 2024 and 28 February 2025 [8].

HRSV is taxonomically classified within the genus *Orthopneumovirus*, family *Pneumoviridae*, harbouring a non-segmented, single-stranded, negative-sense RNA genome of ~15.2 kb, encapsidated within an enveloped virion [9]. Amongst the encoded viral proteins, the attachment (G) and fusion (F) major surface glycoproteins mediate cellular binding, entry and infection [10]. Both G and F elicit immune responses; however, F represents the majority of the primary antigenic determinants for virus-neutralizing antibodies [11]. The G gene shows the most variability in the genome with more than double the number of SNPs normalized by gene length compared to other genes [12]. The G gene also contains the highest number of aa substitutions normalized by protein length [13] and hence is the only gene showing strong evidence of positive selection [12]. Consequently, monoclonal antibodies and vaccines have targeted typically the relatively more conserved F glycoprotein. However, recent evidence suggests these interventions could increase the selective pressures on the F gene, with the potential to reduce the efficacy of such interventions over time [14], as has been observed for other viruses, e.g. severe acute respiratory syndrome coronavirus 2 (SARS-CoV-2) [15,16].

HRSV is classified into two major antigenic subgroups, HRSV-A and HRSV-B, distinguished by mono- and polyclonal antibodies targeting the G protein [9]. Recent studies have characterized such subgroups further into phylogenetically separable lineages according to genetic distances to other lineages using whole-genome sequences [17]. Prior studies have presented conflicting data regarding the clinical severity associated with each HRSV subgroup: nonetheless, these frequently co-circulate in the winter in countries with temperate climates with alternating seasonal patterns of predominance [2,9].

With a significant burden on public health in terms of both morbidity and mortality, HRSV has been a notifiable disease in Ireland since 2012 [18]. The introduction of non-pharmaceutical interventions (NPIs) during 2020 and 2021 to interrupt SARS-CoV-2 transmission was anticipated to, and did, reduce the circulation of other respiratory viruses, including HRSV. Subsequently, however, in the 2022–2023 and 2023–2024 HRSV seasons, a disproportionately high number of HRSV cases were reported in Ireland with large numbers of hospitalizations arising from seasonal HRSV outbreaks, indicating more complex viral transmission dynamics and epidemiology [19,20].

To clarify the potential effects of NPIs during coronavirus disease 2019 (COVID-19) on HRSV genetic diversity and to establish a genomic epidemiological baseline prior to the implementation of more widespread active and passive immunization measures, we have analysed the genomic diversity of HRSV obtained from clinical samples collected both before (2015–2019) and after (2022–2024) the COVID-19 pandemic. We have assessed the classification based on both the HRSV G gene ectodomain and complete genome sequences to assess viral diversity and shown the former to be an acceptably robust approach. We also demonstrate the disappearance of genetic lineages, lineage replacement events and a newly emerging, post-pandemic lineage (A.D.1.4) within HRSV-A, which circulated widely in Ireland and other European countries in late 2023. These findings highlight the need for robust and rapid pathogen genomics to better inform public health policies with the availability of new vaccines and monoclonal antibody countermeasures.

## Methods

### Sample and data collection

Prior to the 2023–2024 respiratory season, no national framework for HRSV genomic surveillance was in place; therefore, samples preceding this season were chosen for sequencing based on surrogate markers of viral titre from diagnostic assays, including multidimension detection (MDD) values ≥300 or Ct ≤25, and availability of residual volume in the sample. Thus, all eligible HRSV-A-positive samples were included, and we performed a random selection of a comparable number of HRSV-B samples with the same criteria. From the 2023–2024 season, the Health Protection Surveillance Centre (HPSC) issued a request for hospitals to refer HRSV-positive samples to the National Virus Reference Laboratory (NVRL) for characterization and to maximize genome coverage; the same high input viral titre criteria for molecular diagnostic assays were employed. Clinical samples were collected and sent to the NVRL for laboratory diagnosis by molecular methods for patients presenting with symptoms related to HRSV across Ireland between 2015 and 2024. Data were compiled and summarized from publicly available national disease surveillance reports, produced by the HPSC (https://www.hpsc.ie/notifiablediseases/annualidstatistics/) and downloaded between October 2017 and May 2024 (https://respiratorydisease-hpscireland.hub.arcgis.com/, downloaded on May 16). Reference genome sequences for the clades were downloaded from HRSV Genotyping Consensus Consortium repository (https://github.com/rsv-lineages) for HRSV-A (*n*=246) and -B (*n*=145) (accession numbers listed in Table S1, available in the online Supplementary Material) [17].

### Sample extraction and storage

Nasopharyngeal samples were extracted using the Roche MagNA Pure 96 DNA and Viral NA Small Volume kit according to the manufacturer’s instructions and eluates stored at −80 °C prior to use.

### Molecular diagnostics

Respiratory samples received for routine screening were tested using the NxTAG Respiratory Pathogen Panel+SARS-CoV-2 assay (Luminex), which identifies both HRSV-A and -B subgroups. Additionally, samples referred to the NVRL for further characterization were tested using a laboratory-developed reverse transcription quantitative PCR (RT-qPCR), based on previously described protocols [21,22], which also allowed discrimination between HRSV-A and -B. Specimens with a viral titre yielding results of MDD ≥300 (NxTAG) or Ct ≤25 (RT-qPCR) were considered eligible for sequence analysis.

### HRSV G gene sequencing

Laboratory-confirmed HRSV-positive samples from the period 2015–2019 were sequenced by standard Sanger approaches with nested PCR targeting the genomic region corresponding to the G protein ectodomain (aa 78 to the end of the protein) [23], using primer sequences provided in Table S3, covering 756 out of 966 nt of the gene. Sequences were deposited in GenBank with accession numbers PP957934–PP958215, and metadata for these samples is provided in Table S3.

### HRSV whole-genome sequencing

The reference genome alignment for a diverse group of target sequences was compiled from available complete and nearly complete genomes for HRSV-A and -B in GenBank. Sequences were organized into clusters with *usearch* software with the *cluster_fast* option after sorting by length (http://www.drive5.com/usearch/). Clustering at 2% identity was employed, and representative sequences were chosen for each cluster to avoid the overrepresentation of common strains or underrepresentation of genetic outliers. Cluster sizes were tracked with the *sizeout* option, resulting on eight clusters for HRSV-A of 461, 243, 114, 85, 62, 42, 15 and 7 sequences, represented by MW582528, KJ643561, KU950673, FJ948820, KJ643484, MG642060, MW768128 and GU591767, respectively. Analogously, HRSV-B yielded four clusters of 810, 75, 15 and 5 sequences, represented by KP317952, KF893260, KP317923 and MN167850, respectively. Cluster-representative sequences were aligned for HRSV-A and -B and used as input in *primalscheme* (https://primalscheme.com/) to design two sets of primers for tiling amplicon multiplex PCR. The resulting primers were aligned to the alignment of all representative cluster sequences with MAFFT [24] using the *addfragments* option, to allow inspection. Alternative primers were added at positions where too many differences were found with the primer-binding sites in sequences representative of the smaller clusters and for missing fragments, based on the sequence of the overlapping part of neighbouring fragments. Laboratory-confirmed HRSV-positive samples received and stored between 2021 and 2024 were processed for whole-genome sequencing (WGS) with a next-generation sequencing approach employing the RSV subgroup-specific oligonucleotide primer pools (Metabion, Germany) described in Tables S2 and S3 upstream of an Illumina DNA library preparation as described downstream of the tagmentation step [25] on the Illumina NextSeq 1000 with a 2×150 cycle run. Assembly of the genomes was performed using the INSaFLU platform (https://insaflu.insa.pt/) that assembles consensus sequences by reference mapping of the reads, with reference for HRSV-A and -B as EPI_ISL_412866 and EPI_ISL_1653999, respectively. Sequences were made publicly available at GenBank (PP969941–PP970173) and also in GISAID (see accession numbers and reference coverage details in Table S4). Additionally, we further validate bioinformatically the genome assemblies to verify the presence of nt duplications in the G gene in both subgroups [26].

### Phylogenetic analysis

Multiple sequence alignments were performed with MAFFT [24]. Phylogenetic trees corresponding to multiple sequence alignments of datasets with (i) G gene and (ii) genome sequences were inferred by maximum-likelihood approaches with IQ-TREE version 2.3.1 [27] and the support for the topology tested with Shimodaira-Hasegawa-like approximate likelihood ratio test and ultra-fast bootstrap with 10^4^ replicates and considering support for branches with values >80% and >95%, respectively. The lineages of sequences were assigned with Nextclade (https://clades.nextstrain.org/) using hRSV/A/England/397/2017 (GISAID: EPI_ISL_412866, GenBank: PP109421.1) and hRSV/B/Australia/VIC-RCH056/2019 (GISAID: EPI_ISL_1653999, GenBank: OP975389.1) as references for HRSV-A and -B, respectively.

### Phylogenetic compatibility matrix

The divergence across different HRSV genomic regions and its impact on the phylogenetic relationships amongst sequences was assessed in a sliding window fashion, with window lengths of 500 nt and a step of 100 nt. Pairwise evolutionary distance was estimated for each window under a Tamura–Nei evolutionary model and used to infer a neighbour-joining phylogenetic tree for comparison against trees corresponding to other windows in a compatibility matrix, similar to the approach described previously by Carr *et al.* [28], where the compatibility of two windows is defined as the normalized Robinson–Foulds distance [29] between the corresponding trees. Thus, the compatibility reflected how similar are the inferred phylogenies for any two genome windows ranging from 1 (identical) to 0 (completely dissimilar).

### Graphical and statistical analyses

Data analysis and graphic representations were performed with R packages and scripts available upon request. *χ*^2^ tests were used to compare frequencies of lineages per year.

## Results

### HRSV cases in Ireland in 2017–2024

**Fig. 1.**
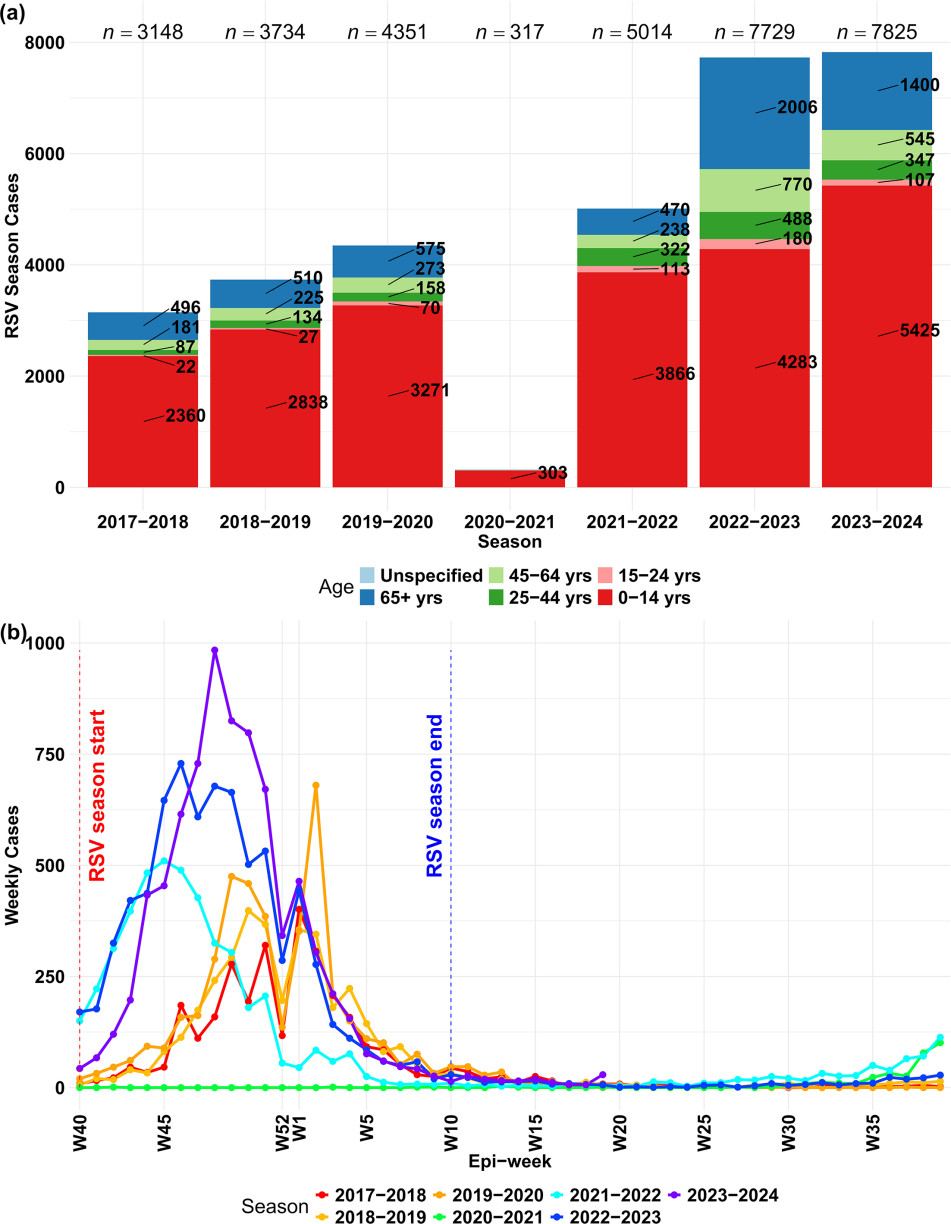
HRSV cases reported between October 2017 and May 2024. (**a**) The total number of HRSV cases (vertical axis) reported in Ireland per HRSV season (horizontal axis). The colours for the age ranges of the patients are shown in the legend at the bottom of the panel. (**b**) The details of weekly counts (vertical axis) collected by surveillance programmes per week (horizontal axis). The start (red dotted line) and end (blue dotted line) of the HRSV seasons in Ireland are highlighted. The HRSV seasonal series of cases per week are coloured as the legend at the bottom. Data source: RSV notification data, HPSC (https://respiratorydisease-hpscireland.hub.arcgis.com).

Publicly available epidemiological data of total HRSV laboratory-confirmed cases reported weekly between October 2017 and May 2024 (*n*=344 weeks) reflect a sharp increment in the last two HRSV seasons (Fig. 1a). Reported cases reflected the most affected groups, prior to and after the COVID-19 pandemic, corresponding to children under 14 years and adults ≥65 years, consistent with prior reports [1]. Furthermore, the combination of these age groups significantly declined from 90% (2856/3148) of cases in the season 2017–2018 to 87% (6825/7825) in the season 2023–2024 (*P*=3.1×10^−7^, *χ*^2^ test) (Fig. S1). It is noteworthy that the number and age distribution of HRSV recorded infections in the season 2020–2021 were likely affected by the impact of NPIs in place to reduce the transmission of SARS-CoV-2 and potentially by an overrepresentation of severe HRSV infections in young children under 15 years of age, through passive surveillance systems during that period.

The HRSV epidemic season is typically defined in Ireland between week 40 and week 10, i.e. from early October to the second week of March of the following year; however, we observed a notable number of HRSV cases accumulating earlier (Fig. 1b) than expected in 2021–2022 and 2022–2023. Following the COVID-19 pandemic, the apparent shift of the beginning and the peak of HRSV seasonality, in combination with the increase in the number of reported cases, could be attributed to the following factors: an increase in the number of susceptible individuals within the population, changes in patterns of healthcare-seeking behaviour amongst individuals experiencing respiratory symptoms and the impact of more widespread access to molecular testing for respiratory viruses in hospital diagnostic laboratories. A similar shift in HRSV epidemiological curves after discontinuation of NPIs has been observed in the USA with a suggestion that the coming winter seasons will see a return to the previous seasonality [30].

### Assessment of lineage-informative loci in HRSV genomes

**Fig. 2.**
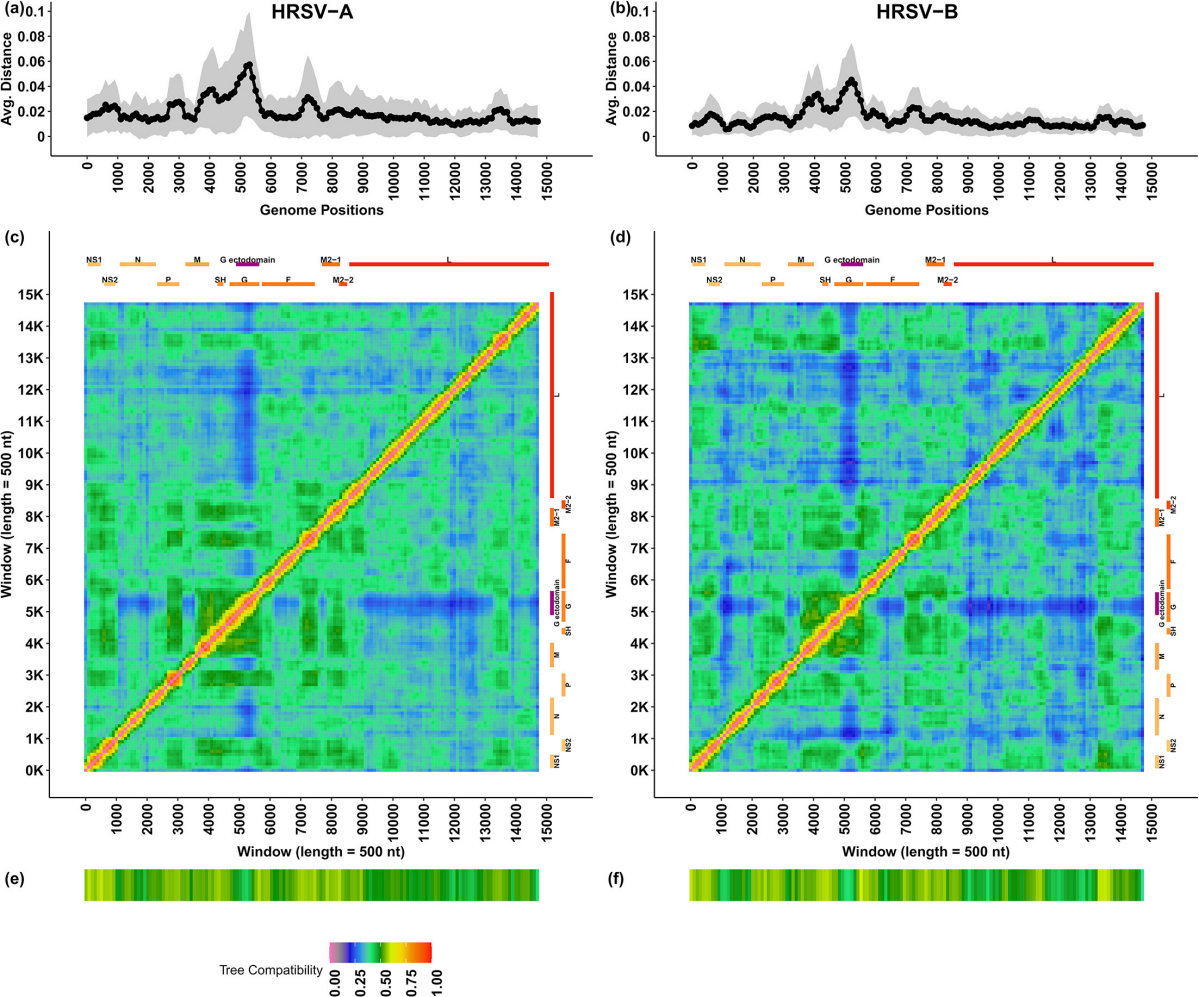
Distribution of genomic diversity along the HRSV-A and HRSV-B genomes. The visualized data correspond to windows (length=500 nt) from sliding window analyses along the multiple sequence-aligned reference genomes of HRSV-A and HRSV-B. The horizontal axes represent the genome positions of the windows. (**a, b**) The average evolutionary distance (under a TN93 model) per window represented by the black series. The vertical axis represents the evolutionary distance. The grey shadow shows one sd above and under the average for the windows. (**c, d**) Phylogenetic compatibility matrices comparing the compatibility between phylogenetic trees corresponding to the windows of the genome. The vertical and horizontal axes represent the genome positions of the windows being compared. The compatibility is coloured according to the legend at the bottom of the panel. The gene annotation corresponding to the windows is shown at the top and right of the heatmap, including G ectodomain. (**e, f**) Phylogenetic compatibility of each window tree against the tree of the corresponding whole genome coloured following the legend at the bottom of the panel.

We assessed the evolutionary distance amongst HRSV reference genome sequences of both viral subgroups using a sliding window approach (Fig. 2a, b). The highest nt sequence diversity amongst sequences was located in the G gene as 0.057±0.042 and 0.045±0.030 for subgroups A and B, respectively (Fig. 2a, b). The G gene had lower phylogenetic compatibility to other genomic regions (Fig. 2c, d) and to the phylogenetic tree of the whole genome (Fig. 2e, f). The locus encoding the G gene is suggested to be less constrained by structural and functional interactions potentially attributable to humoral selective pressures [31], therefore representing an informative sequencing target for tracking HRSV lineages and their epidemiological distribution (Fig. 2c, d). It is noteworthy that despite the epidemiologically informative nt variability of the G gene, G is not the target for HRSV prophylaxis, thus limiting the interpretation of surveillance based solely on this gene. G gene evidenced an average pairwise distance amongst reference sequences of 0.05±0.04 and 0.04±0.03 in the HRSV-A and -B datasets, respectively, whilst the average pairwise distance at genome sequence level was 0.02±0.02 and 0.01±0.01. The nt diversity in the G gene observed in both the sliding window and pairwise distance gene analyses was consistent with prior reports described above, indicating that this gene is under positive selection in HRSV [12,13]. Notably, this genetic variability has been employed previously for taxonomic designation of groups and genotypes [9], allowing the epidemiological tracking of circulating strains and outbreaks. In contrast, the F gene contains lower genetic diversity, highlighting its sequence conservation, relative to the G gene, and diverges at a comparable proportion to the rest of the genome. The higher mutation rate within the G gene has been suggested to indicate a potential role in the viral adaptive strategies [32].

**Fig. 3.**
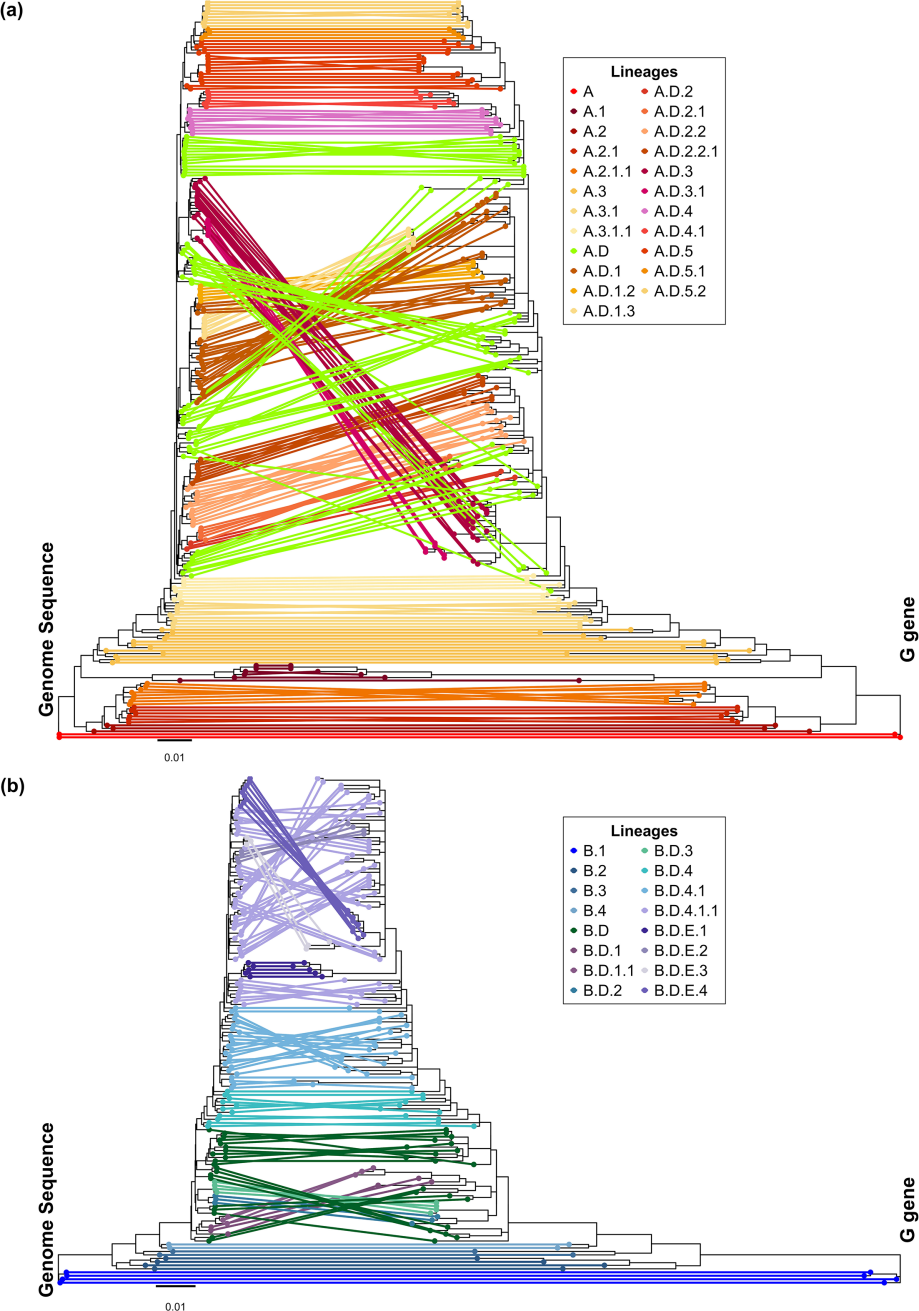
Comparison of tree topologies for HRSV reference sequences. In panels (**a**) and (**b**), the left-hand tree shows the phylogenetic tree inferred for the complete genome sequences of (**a**) HRSV-A and (**b**) HRSV-B and the right-hand phylogenetic tree inferred for the corresponding G target sequences. Sequences in both trees and lines connecting them are coloured according to the lineages as shown in the legend at the right of each panel.

The virus classification consistency was assessed by comparing lineages assigned using the G locus against lineages assigned using whole-genome sequences of the reference dataset. This showed 96% (235/246) and 99% (144/145) consistency in the assigned lineages to each sample based on G ectodomain and complete genome sequences of HRSV-A and -B, respectively. Amongst the inconsistencies, there were nine HRSV-A reference sequences and only one HRSV-B misclassified (details are shown in Table S1). Although phylogenetic trees inferred using the G ectodomain and complete genome sequences showed different topologies in the relationship between lineage clades (Fig. 3a, b), the sequences within the same lineage remained consistently associated. The different phylogenetic topologies between the G gene and genome phylogenies were attributed to the distribution of more lineage-specific mutations in other genomic loci. In addition, the average intra-lineage evolutionary distances confirmed the high similarity of sequences under the same lineage and higher differences to sequences in other lineages (Fig. S3A, B). The G ectodomain was found to be a reliable target for sequence classification, comparable to the whole G gene, as well as the L and F genes in relation to their sequence length (Fig. S2A, B). This conclusion was supported by tree distances to the genome phylogenetic tree (Fig. S2C, D), the shared phylogenetic information (Fig. S2E, F) and the mutual clustering information with the complete genome sequences (Fig. S2G, H). Therefore, we infer that the lineage classification achieved by the G ectodomain is comparable to the lineage classification based on the whole-genome sequences.

### Epidemiology of HRSV cases in 2015–2024 in Ireland and Europe

**Fig. 4.**
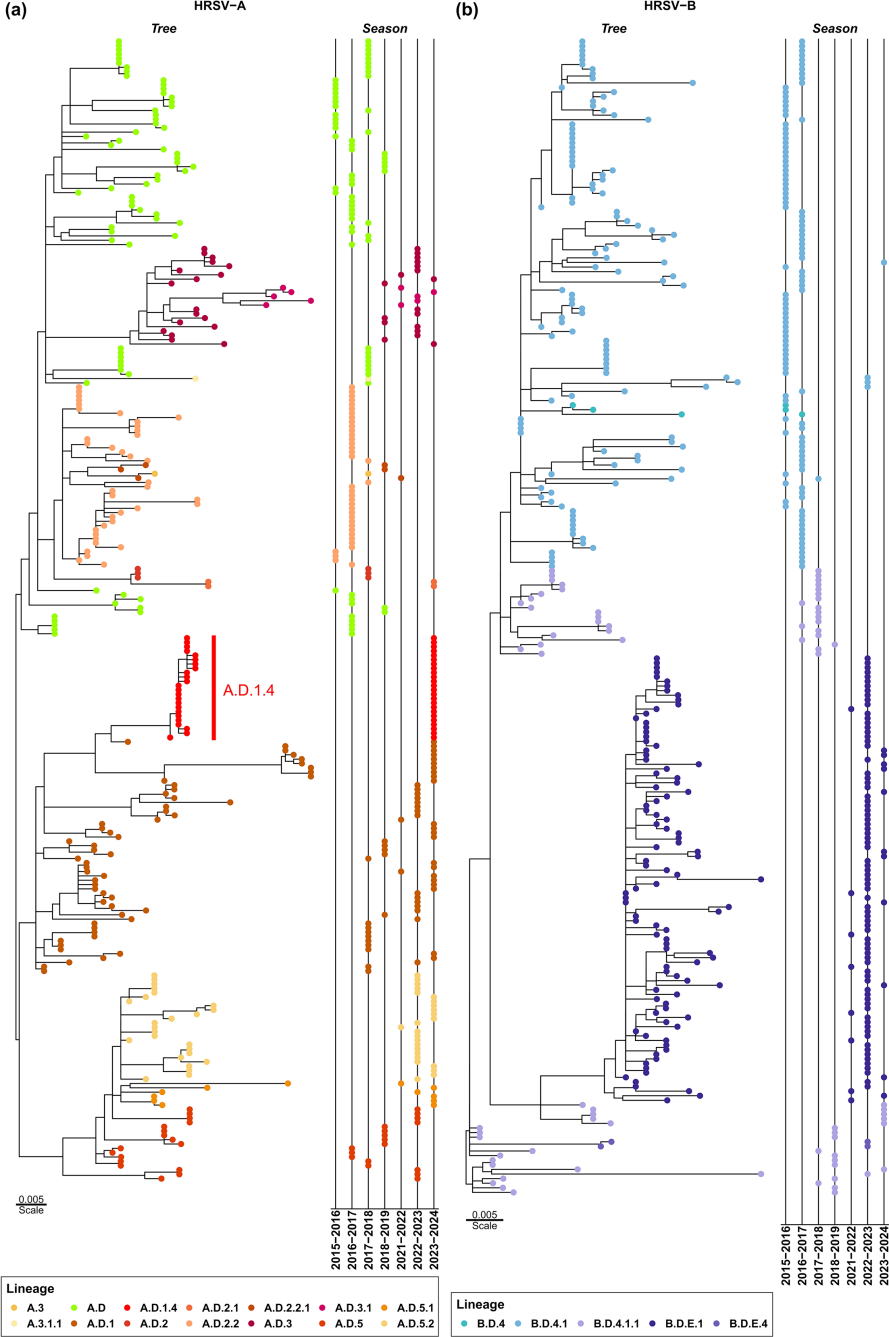
G gene phylogenetic trees of HRSV cases in Ireland between 2015 and 2024. The left facets of the panels show the phylogenetic trees for (**a**) HRSV-A and (**b**) HRSV-B. The right facets of the panels show the sampling season, shown at the bottom of the facet, for the corresponding horizontally aligned samples. Tips are coloured according to the assigned lineages shown on the legend at the bottom of the panels. The tree scales represent the branch lengths in base substitutions per site. Tips of samples under the new lineage A.D.1.4 are highlighted with a vertical red bar in (**a**).

The available Irish HRSV lineage data in 2015–2019 corresponds to lineages assigned, employing G gene sequencing. Such partial sequences were compared against lineages detected in Ireland in 2021–2024 and assigned with complete genome sequences for both viral subgroups (Fig. 4). In HRSV-A, clusters of samples in years 2022 and 2023 indicated decreased viral lineage diversity (Fig. 4a). Furthermore, HRSV-B lineage analysis demonstrated the prevalence of a single lineage during the same period (Fig. 4b). Nevertheless, comparisons of pairwise evolutionary distances between the different sampling seasons supported a significantly greater evolutionary diversity in the G locus for both groups in samples from 2022 to 2023 and 2023 to 2024 than in samples previous to 2020 (*P*<0.05, *χ*^2^ test) with the exception of HRSV-B in 2016–2017, which showed the most diverse range of genetic evolutionary distance (Fig. S4). The HRSV sequence diversity in the recent seasons suggested a diversification of the novel lineages. In addition, the decreased co-circulation of the 2015–2019 lineages (Fig. 4) likely explains the observed increased average pairwise evolutionary distances (Fig. S4). Notably, for both HRSV subgroups, changes in the lineage prevalence patterns were observed following the comparison of the periods preceding and following the COVID-19 pandemic. The sequences from the 2021 to 2024 period clustered together under a single clade for 82% (*n*=101/123) and 88% (*n*=97/110) of sequences in the HRSV-A (Fig. 4a) and HRSV-B (Fig. 4b), respectively.

**Fig. 5.**
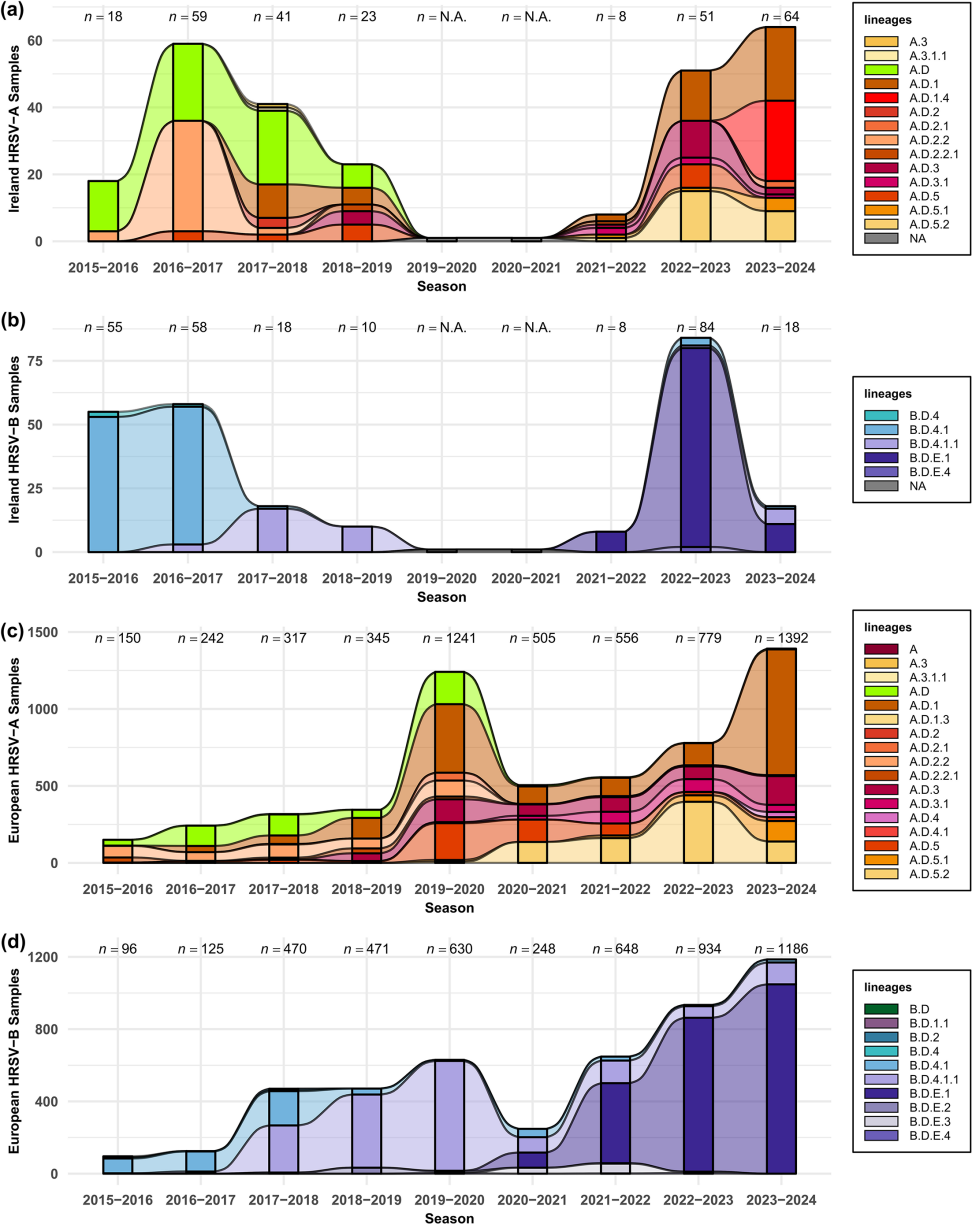
HRSV clades detected in Ireland and Europe between 2015 and 2024. The total number of cases sequenced (vertical axes) per HRSV season (horizontal axes) and stratified and coloured per detected clades as shown in the legend at the right of each panel. (**a**) and (**b**) show the data for HRSV-A and HRSV-B, respectively, in Ireland, while (**c**) and (**d**) show the data for HRSV-A and HRSV-B in the rest of Europe.

The predominant lineages per year were further explored for HRSV-A (Fig. 5a) and HRSV-B (Fig. 5b). In both subgroups, we found statistically significant changes in the frequencies of predominant lineages between years preceding the COVID-19 pandemic (2015–2019) and the first years after the NPIs introduced during the pandemic were lifted (2022–2024). In HRSV-A, significant changes in the number of cases per lineage were seen (*P*<3×10^−33^, *χ*^2^ test). The first period was dominated by lineages A.D, A.D.2.2 and in lesser degree A.D.1; in the second period, the predominance changed to A.D.1 with a larger percentage (32%, *n*=123) of the cases, A.D.5 phylogenetically derived lineages and a novel lineage A.D.1.4 representing 20% of cases. Similarly, the HRSV-B showed a significant predominance change (*P*<9×10^−45^, *χ*^2^ test) from B.D.4.1 and later B.D.4.1.1 predominant in the pre-pandemic period, to a predominance of B.D.E.1 after the pandemic.

The changes in the lineage predominance circulating prior to and following the COVID-19 pandemic indicated the replacement of circulating viral genetic lineages in Ireland. To assess this interpretation, lineages circulating in Europe (excluding Ireland) were compared over the same time period, by analysing the whole-genome sequences reported in GISAID for both HRSV-A (Fig. 5c) and HRSV-B (Fig. 5d and Table S4). Notably, this separate international analysis also yielded significant results for both subgroups when comparing similar periods 2015–2019 and 2022–2024 (*P*<9×10^−100^, *χ*^2^ test). The higher number of sequences in the European dataset contextualized the observations from the national dataset, reflecting very similar trends, with an increase in the prevalence of A.D.1 (44%, *n*=961/2171) and A.D.5.2 (25%, *n*=536/2171) for the HRSV-A subgroup and a change in prevalence of B.D.E.1 in HRSV-B in recent years (90%, *n*=1900/2120). Moreover, this regional context provided a plausible explanation for the origin of novel HRSV-B lineages circulating in Ireland after the COVID-19 pandemic. In addition, the classification system employed allows diversity to be measured using the same criteria for lineage definition in both subgroups. HRSV-A showed a broader distribution of co-circulating lineages, whilst HRSV-B not only had an overrepresentation of a single lineage but also a lower overall number of co-circulating lineages (Fig. 5). It is worth considering that any potential bias due to WGS was also present for both subgroups in the past. The trends in diversity observed now align with those seen before the increase of available sequences, suggesting that the observed patterns are not solely a consequence of improved sequencing methods. However, continued surveillance is needed to determine whether this represents a transitional phase, with HRSV-A potentially adopting a diversity profile similar to HRSV-B or vice versa.

### Novel HRSV-A lineage circulating in Ireland and internationally

**Fig. 6.**
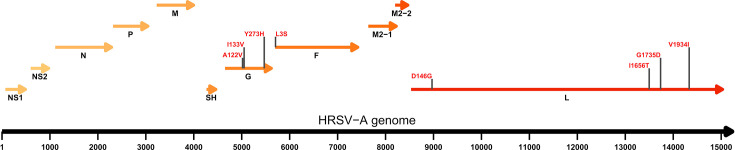
aa substitutions present in the novel HRSV-A A.D.1.4 lineage. Annotated HRSV-A genome representation with the distribution of genes and aa substitutions distinguishable from other lineages. The horizontal axis corresponds to genome positions, and the predicted products are annotated as arrows. Substitution positions are annotated with vertical black lines and the aa changes in red, indicating the reference aa, position relative to the protein and the aa substitution.

Our genomic surveillance allowed the detection and characterization of a novel HRSV-A lineage descendant from A.D.1 (Fig. 4a) under the standardized nomenclature criteria recently established by the RSV Genotyping Consensus Consortium [17]: the novel lineage must present ≥5 aa mutations distributed amongst multiple proteins and form a monophyletic, highly supported cluster of multiple sequences. We identified 24 HRSV-A sequences classified within the A.D.1 lineage with at least 8 characteristic aa substitutions of unknown functional effect (Fig. 6): 3 substitutions in the G protein, 1 substitution in the F protein and 4 substitutions in the L protein. Experimentally characterizing the functional effects of such aa substitutions was beyond the scope of the present study; however, we mapped their locations to the functional domains of the proteins. The three substitutions in G protein, A122V, I133V and Y273H, reside in the two mucin-like domains (domain I: 66–164 and domain II: 198–298) [23], which could potentially impact immune evasion [33]. Substitution in F protein, L3S, located in the N-terminal domain could have effects on protein processing as the section is proteolytically removed in the mature F protein and corresponds to the signal peptide [34]. Finally, four aa substitutions in L protein are located in multiple domains associated with virus replication and infection: notably, D146G in the RNA-dependent RNA-polymerase domain; I1656T and G1735D are located in the connector domain between capping and methyltransferase domains, and V1934I is located in the mononegavirus-type S-adenosylmethionine-dependent 2′-O-MTAse [35]. Assessing the impact of these substitutions, if any, requires further experimental characterization. This novel lineage has been formally named A.D.1.4 following the current nomenclature and procedures for the assignment of a new viral genetic lineage. These substitutions as criteria allowed the identification of another 90 instances of this lineage circulating in Europe (*n*=53), Australia (*n*=12), Brazil (*n*=15) and the USA (*n*=10) (Table S5), with 63 of those preceding cases in Ireland.

### Potential effects of diversity on available treatments

The aa conservation of F protein amongst all Irish samples was analysed in 2022–2024 to identify substitutions in the binding sites to the monoclonal antibody nirsevimab in residues 62–69 and 196–212, following the results reported by Wilkins *et al*. [36]. Amongst HRSV-A, 3 out of 124 (2%) sequences showed a substitution affecting site 65 in the F2 subunit from lysine (K) to arginine (K65R) in clade A.D.3.1, which *in vitro* has been reported to result in a <10% fold change in IC_50_ [36]. This substitution has also been observed in sequences of samples from the UK and the USA. HRSV-B exhibited greater divergence in F1 subunit sites, with isoleucine (I) at residue 206 substituted by methionine (M) in 107 out of 111 sequences (I206M), consistent with the observation of this substitution being more prevalent in recent samples globally and *in vitro,* leading to a <10% fold change in IC_50_ [36]. Moreover, sites 209 and 211 also showed substitutions from arginine (R) and asparagine (N), respectively, to glutamine (Q) (5 out of 111 sequences) and serine (S) (8 out of 111 sequences) with these substitutions concurrent in 4 sequences of B.D.4.1, 1 B.D.E.1 showing the R209Q (observed also in sequences from the UK, France and North America), 2 B.D.E.4 (observed in the USA and Australia) and 1 B.D.4.1.1 lineages showing the N211S substitution (widely observed in multiple geographical locations). These substitutions were recently identified and require further characterization to assess any potential impacts on prophylactic efficacy. We performed a similar analysis focused on the palivizumab-neutralizing epitope in F aa residues 258–275, following the results reported by Hashimoto and Hosoya [37]; however, no substitutions were found affecting this region of F protein in either viral subgroup.

## Discussion

Our genomic surveillance revealed that COVID-19 containment measures disrupted the HRSV seasonality during the 2020–2021 season in Ireland, suggesting, unsurprisingly, that these interventions provided protective effects against the spread of HRSV and other common respiratory viruses. However, HRSV seasons 2022–2023 and 2023–2024 showed increases in case numbers, likely due to a growing immunologically susceptible population, waning immunity and enhanced testing capabilities for respiratory infections, all potentially augmented by changes in the healthcare-seeking behaviour of younger populations after the COVID-19 pandemic. These periods also showed changes in circulating viral genetic lineages, with marked increases in A.D.5.2 in HRSV-A and B.D.E.1 in RSV-B, reflecting a regional shift in lineage prevalence potentially influenced by an HRSV transmission bottleneck effect during the COVID-19 pandemic. Additionally, we also identified a novel post-pandemic HRSV-A lineage circulating in Ireland and internationally since 2023. Importantly, we have detected HRSV-B strains characterized by two notable substitutions in F protein (R209Q and N211S), which may affect the efficacy of the long-acting monoclonal antibody preparation, nirsevimab.

The current study provides evidence supporting the comparability of HRSV clade classification data based on partial sequencing of a 756-nt segment of the G gene with complete genome sequences in both subgroups, HRSV-A and HRSV-B, consistent with recent findings by Goya *et al.* [38]. Such evidence enabled the comparison and epidemiological analysis of HRSV genetic lineages circulating in Ireland during 2015–2019 against lineages circulating in 2022–2024 following the lifting of NPIs introduced to prevent SARS-CoV-2 transmission.

In addition, our results support the informative role of sequencing the G gene for clade classification and surveillance information; they also demonstrate the value of more comprehensive WGS methods to assess viral divergence across the entirety of the HRSV genome, particularly within the vaccine-relevant F external glycoprotein to assess changes in the frequency of aa substitutions as pharmaceutical countermeasures become available. The criteria for assignment of novel HRSV lineages include the presence of aa substitutions in proteins other than the G protein [17], as potentially novel strains with mutations in other genes could show enhanced transmissibility or exhibit immune escape from current prophylactic measures. Molecular surveillance also enables computational analysis of PCR-based molecular diagnostic testing, characterization of seasonal epidemics and the efficacy of ongoing responses and putative therapies. Such efforts are similar to current influenza surveillance programmes. We identified two substitutions in the F protein of HRSV-B that could affect the efficacy of treatments with nirsevimab, R209Q and N211S, which require further functional characterization to assess their impact.

Also, this genomic surveillance initiative uncovered a novel lineage circulating in Ireland during 2023 named A.D.1.4. This lineage, a descendant from A.D.1, is related to the sequences from a number of other European countries exhibiting the same distinguishable substitutions since 2020. Although the effects of the reported substitutions remain to be experimentally explored, the growing frequency across international borders of a lineage that appeared during the COVID-19 pandemic is consistent with our observations of changes in the HRSV lineage dominance with possible relation to the impact of NPIs on extant viral genetic diversity. These changes in lineage abundance, lineage replacement events and the emergence of new genetic lineages necessitate the continuous revision and updating of primer pools for amplicon-based WGS approaches and HRSV lineage designation to properly quantify and track epidemiological trends and evaluate potential risks, as previously suggested by others [17]. Following from the efforts during the COVID-19 pandemic, improvements in molecular diagnosis, genetic characterisation, reporting, and sequencing increased the annual number of HRSV genome sequences generated allowing more detailed monitoring of lineages in circulation over time. However, caution must be taken when discussing the viral fitness of different lineages, as distinguishing the effects of advantageous substitutions from a founder effect can become difficult, e.g. the effects of the D614G substitution in the fitness of the virus during the COVID-19 pandemic [39]. Further functional characterization is clearly warranted of the novel HRSV lineage we have identified here.

We were limited by the number of samples with enough recoverable genetic material to be sequenced despite the number of yearly cases. Additionally, a stringent Ct threshold (≤25) was employed to maximize the chances of obtaining whole-genome sequences, limiting the sequencing rate to 1–2% of the total number of Irish cases, which may have influenced the detection of HRSV lineages. Cases spanning the period 2020–2021 were not stored for sequencing as the workload demands and focus on SARS-CoV-2 precluded the storage and subsequent analysis of HRSV lineage distribution during this period. This limitation was partially addressed by analysing the lineages circulating in the European region.

In conclusion, this study represents the first comprehensive analysis of HRSV lineages circulating in Ireland, spanning pre- and post-COVID-19 pandemic periods, demonstrating notable shifts in viral genomic diversity likely attributable to the widespread institution of NPIs between 2020 and 2021. Our data also offer an important baseline for future analyses of HRSV genomic diversity in the context of anticipated novel preventive modalities and a framework to monitor for the emergence of variants with the potential to impact treatment efficacy, or vaccine escape.

## supplementary material

10.1099/mgen.0.001379 Uncited Supplementary Material 1.

## Abbreviations

COVID-19: coronavirus disease 2019
HPSC: Health Protection Surveillance Centre
HRSV: human respiratory syncytial virus
MDD: multidimension detection
NPIs: non-pharmaceutical interventions
NVRL: National Virus Reference Laboratory
RT-qPCR: reverse transcription quantitative PCR
SARS-CoV-2: severe acute respiratory syndrome coronavirus 2
WGS: whole-genome sequencing
