# Supplementary material for: Human respiratory syncytial virus genetic diversity and lineage replacement in Ireland pre- and post-COVID-19 pandemic
Source: Microb Genom. 2025 Mar 17;11(3):001379. doi: 10.1099/mgen.0.001379 (PMC11914048; doi:10.1099/mgen.0.001379)
Supplement: Uncited Supplementary Material 1. [file mgen-11-01379-s001.pdf]

Supplementary Materials

Supplementary Tables 1 to 5 are datasets of metadata publicly available at: https://doi.org/10.5281/zenodo.12797565

Supplementary Table 1. Metadata of NextClade Reference Sequences publicly available at https://github.com/rsv-lineages.

Supplementary Table 2. The whole-genome sequencing library preparation protocol template with concentrations of the oligonucleotide primers and reagents for amplification and sequencing.

Supplementary Table 3. Oligonucleotide primers for RT-PCR amplification and sequencing protocols targeting the locus encoding the G protein ectodomain and the whole genome sequencing.

Supplementary Table 4. Irish RSV Sequence Details. Details of the sequences generated in our study are provided with respect to the GenBank accession number, the GISAID accession number, reported collection RSV season and the Nextclade assigned taxonomical clade.

Supplementary Table 5. International RSV Sequence Details. A list of European RSV sequences downloaded from GISAID on May 2024 (https://gisaid.org/) is provided together with sequences identified as part of the A.D.4.1 lineage in July 2024, with details of the sampling RSV season, the accession number, country of collection and lineage assigned by NextClade.

Supplementary Figures.

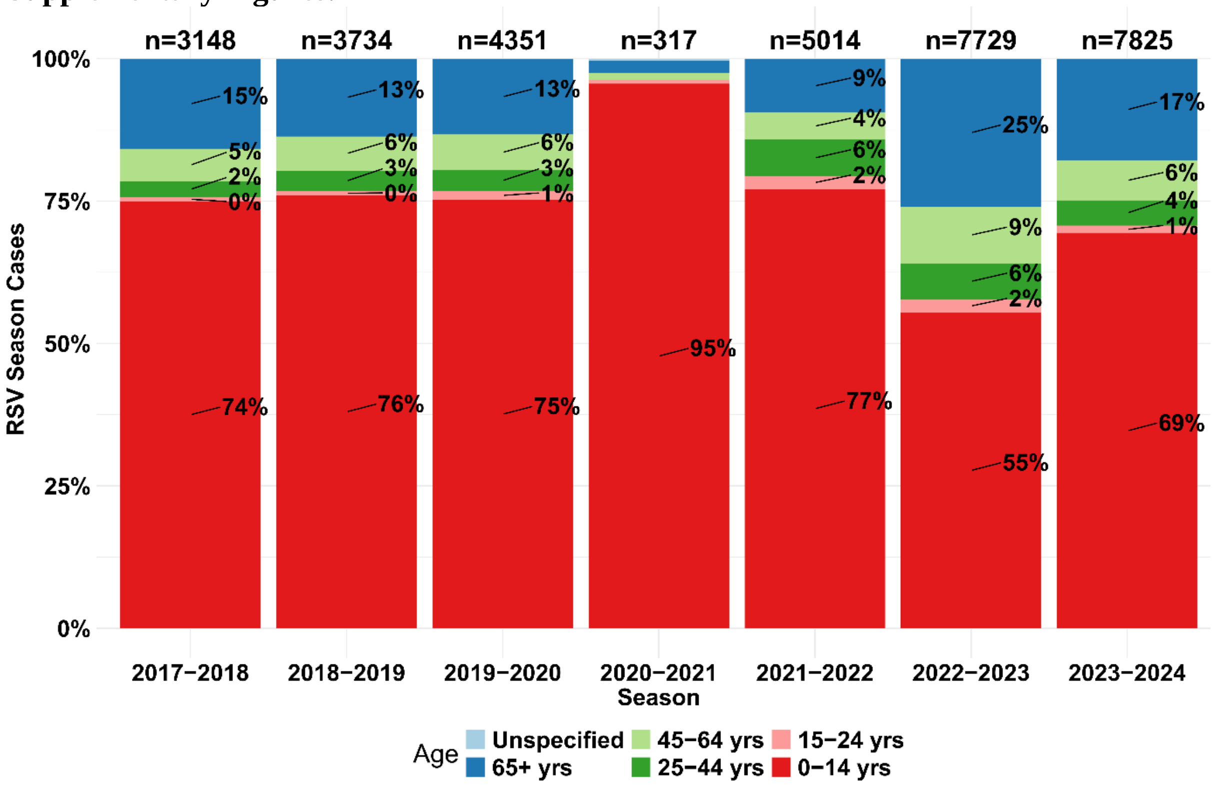

Supplementary Figure 1. Proportion per age group of HRSV cases reported between October 2017 and May 2024. The percentage of HRSV cases (vertical axis) reported in Ireland per HRSV season (horizontal axis). The colours for the age ranges of the patients are shown in the legend at the bottom of the panel.

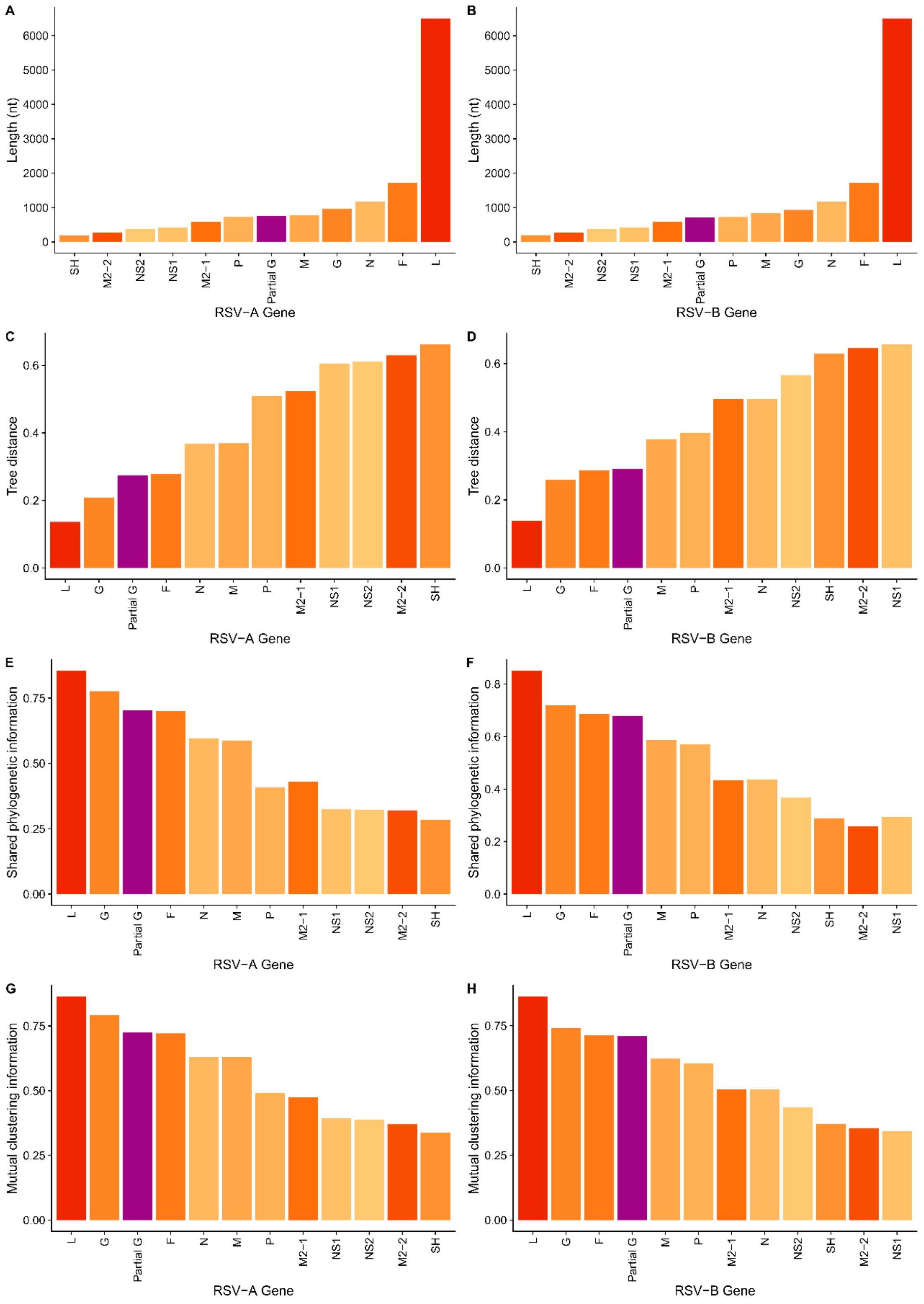

Supplementary Figure 2. Multiple measures of phylogenetic tree consistency between the HRSV whole-genome sequences and HRSV genes. A) and **B)** show the length in nucleotides of genes and the segment partial G sorted from shortest to largest. **C)** and **D)** show the tree distance to the phylogenetic tree inferred with the complete genome sequences sorted from most similar to most dissimilar. **E)** and **F)** show the shared phylogenetic information sorted from the gene with the most shared phylogenetic information to the least. **G)** and **H)** show the mutual clustering information with the most mutual clustering to the left.

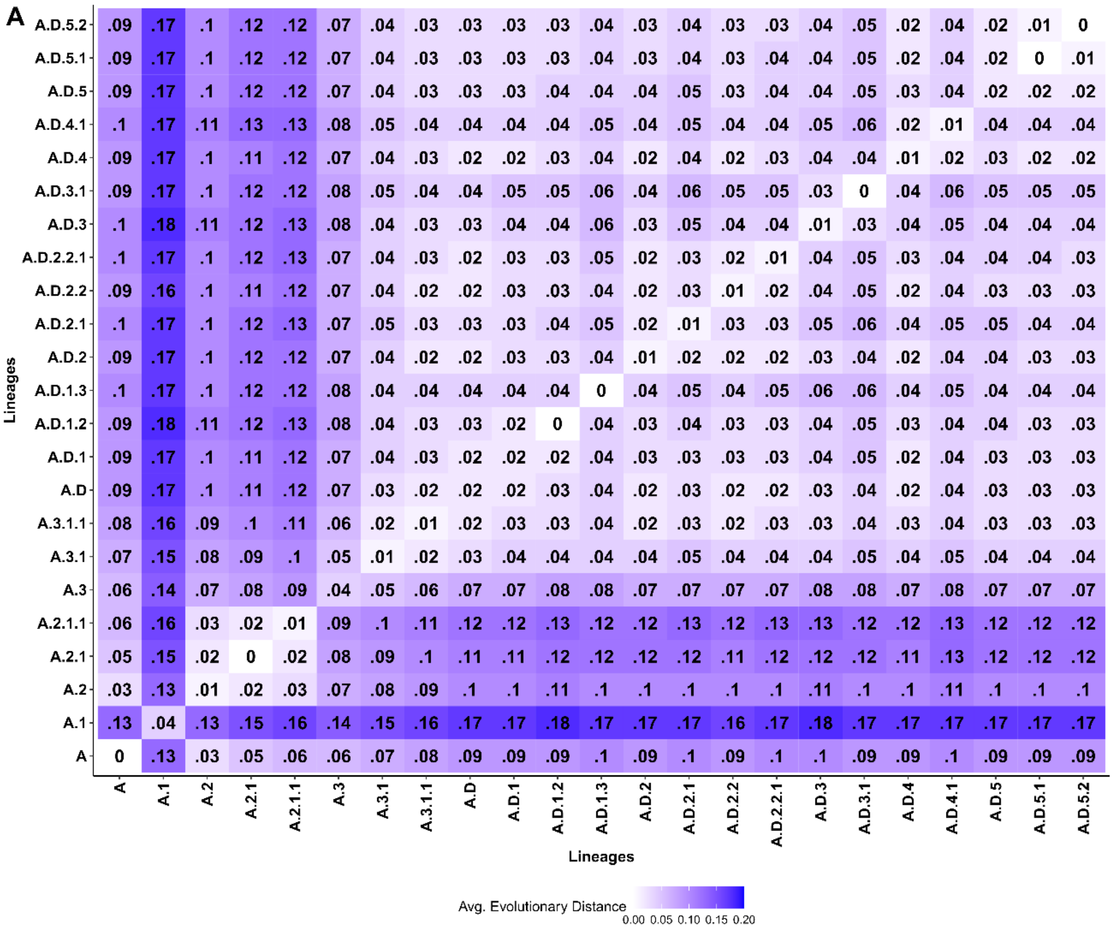

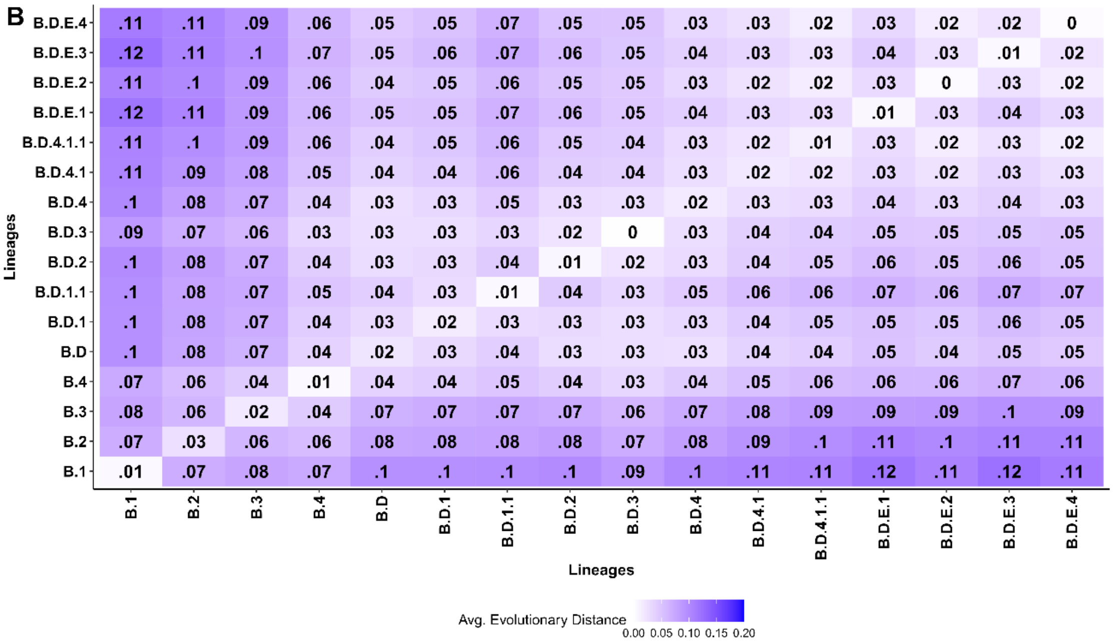

Supplementary Figure 3. Average evolutionary distance in HRSV G gene sequences between lineages. The vertical and horizontal axes show the lineages for **A)** HRSV-A and **B)** HRSV-B, the intersections of the lineages show the average pairwise evolutionary distance between sequences in both lineages and coloured according to the legend at bottom of the panels. With white as the lowest distance and dark blue as the most diverged inter-lineage distances.

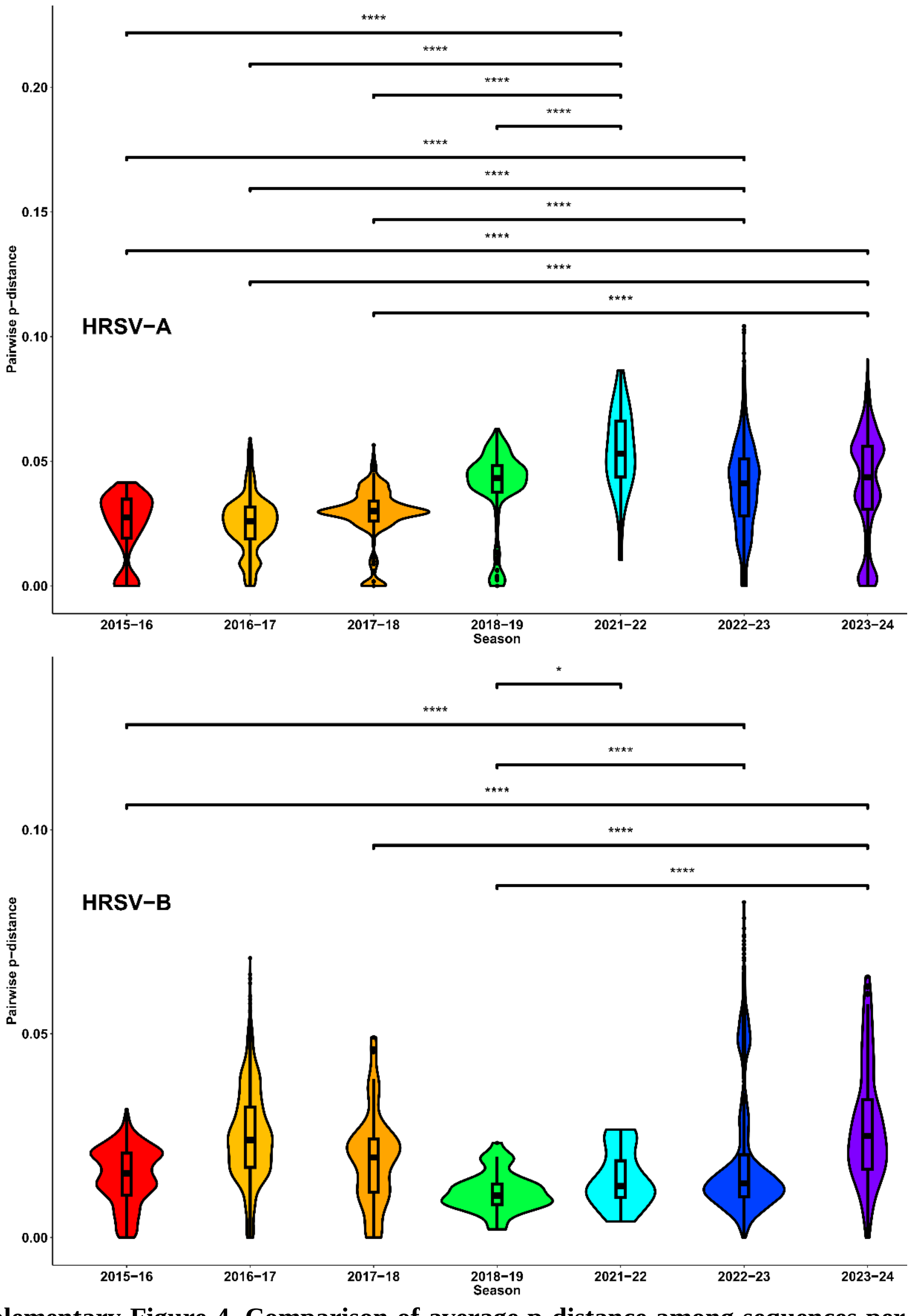

Supplementary Figure 4. Comparison of average p-distance among sequences per year in the HRSV G gene. The means of the distributions were compared to test whether the distributions during 2022 or 2023 had higher mean evolutionary distances than the distributions prior to the SARS-CoV-2 2020 pandemic, under a t-test. Distributions are coloured according to the winter HRSV season. Only significant differences are annotated with a horizontal bar and the statistical significance is coded as *: $p<0.05$, **: $p<0.01$, ***: $p<0.001$, ****: $p<0.0001$.
